# Dual Drainage Total Anomalous Pulmonary Venous Connection: Diagnosis by Cardiac Computed Tomography Angiography

**DOI:** 10.1016/j.jaccas.2025.104891

**Published:** 2025-09-03

**Authors:** José Martín Alanís-Naranjo, Melisa Jessi Inquilla-Coyla, Miguel Fabián Barrera-Colín, Regina de la Mora-Cervantes

**Affiliations:** Cardiovascular Imaging Department, Instituto Nacional de Cardiología Ignacio Chávez, Mexico City, Mexico

**Keywords:** anomalous pulmonary venous return, computed tomography angiography, infant, mixed total anomalous pulmonary venous connection, total anomalous pulmonary venous connection

## Abstract

Among the many variations of total anomalous pulmonary venous connection, the one with dual drainage is extremely rare. This case series presents 3 patients with diverse variations in dual drainage total anomalous pulmonary venous connection thoroughly characterized by cardiac computed tomography angiography.

## Graphical Abstract

**Figure:**
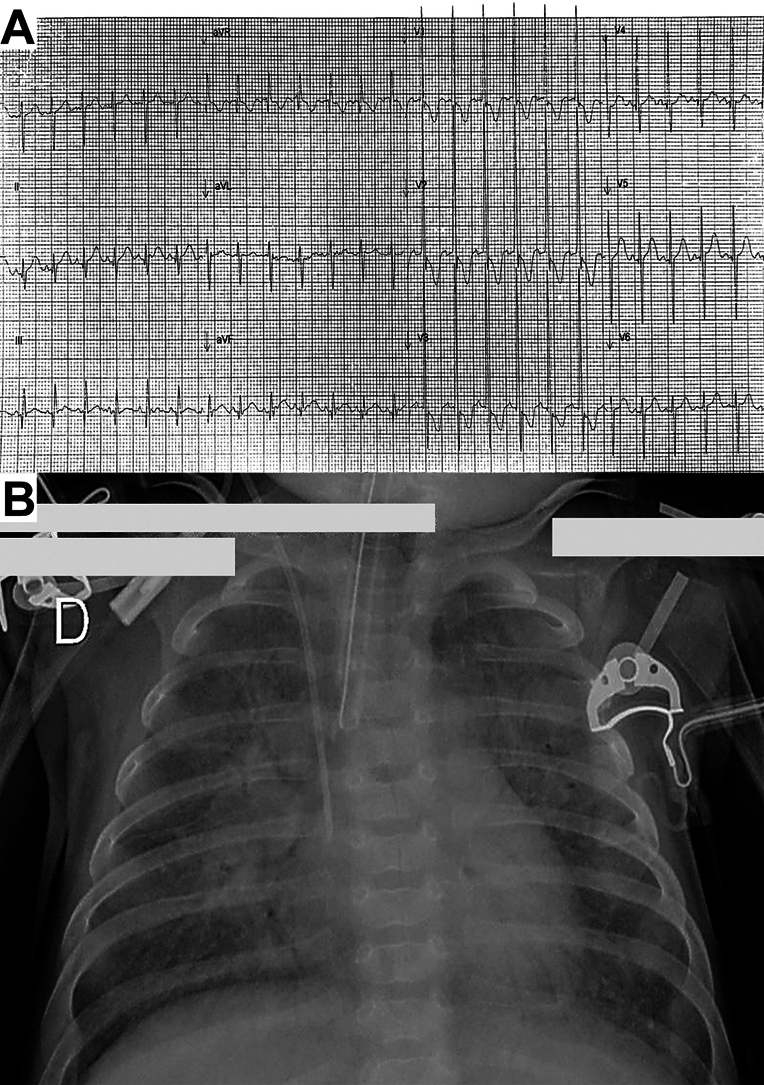

“A pulmonary vein is connected anomalously only when it is attached to a site other than the morphologically left atrium.” —R.H. Anderson^1^

In a total anomalous pulmonary venous connection (TAPVC), the drainage of all pulmonary veins (PVs) is anomalous. This rare condition accounts for 1% to 2% of all congenital heart diseases (CHDs).^2^^,^^3^ The least common variant is a mixed type of TAPVC, occurring in approximately 5% of all patients.^4^ Mixed TAPVC with dual drainage through a common venous confluence is much rarer, with only a few cases reported in the literature.^5^

**Take-Home Messages**

• Dual drainage mixed TAPVC is a rare variant with a fatal outcome when no shunt is present in the left heart chambers.
• The correct preoperative diagnosis and anatomic description of dual drainage mixed TAPVC and associated defects are essential for planning surgical treatment.
• Cardiac computed tomography angiography provides accurate identification of TAPVC types, as well as variations in drainage patterns among mixed variants.
• Surgical mortality remains high in patients with TAPVC who undergo surgical pulmonary vein rerouting and have complex patterns of pulmonary venous connections.

We present a case series describing the clinical presentation and cardiac imaging patterns of 3 patients with different presentations of dual drainage TAPVC.

## Case Series

**Figure 1:**
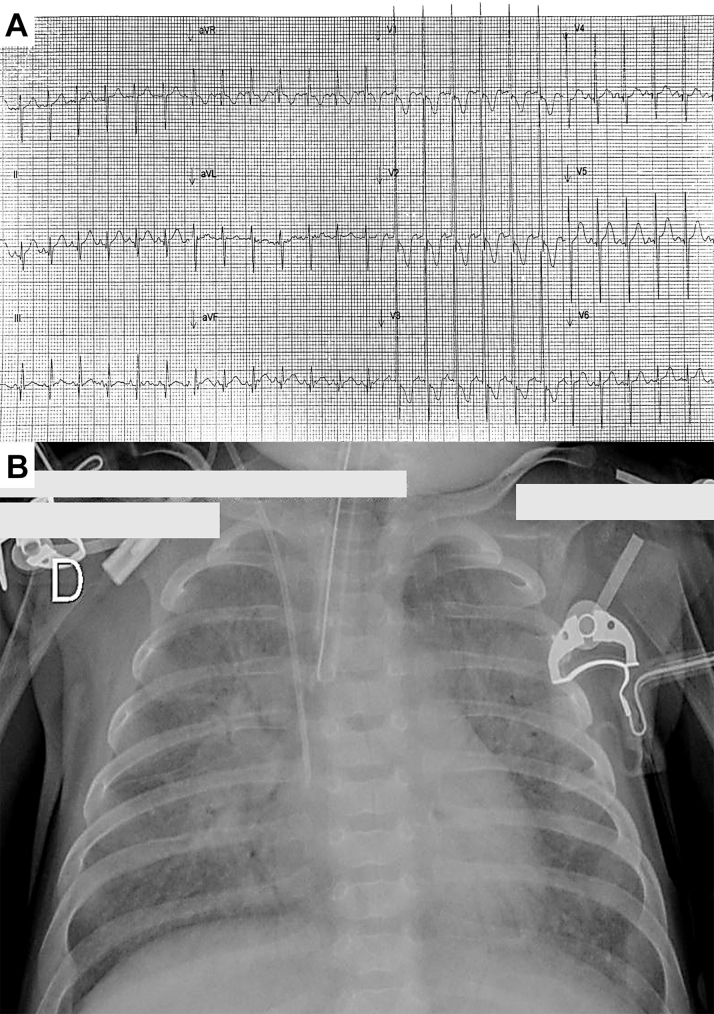
Patient 1 With Dual Drainage Total Anomalous Pulmonary Venous Connection (A) Initial electrocardiogram showing biventricular hypertrophy with right ventricular pressure overload. (B) Chest radiograph on admission showing bilateral pulmonary infiltrates.

**Figure 2:**
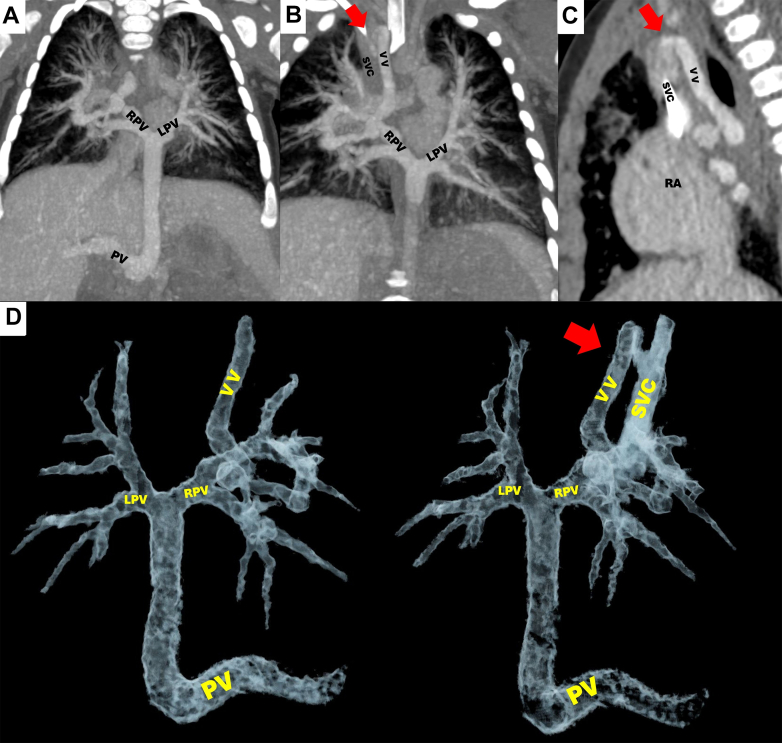
Patient 1 With Dual Drainage Total Anomalous Pulmonary Venous Connection Cardiac computed tomography angiography showing drainage of the right and left pulmonary veins to a common collector, which flows to the portal vein and SVC via a VV (red arrow). The VV receives segmental branches from the right upper and middle pulmonary lobes. (A, B) Coronal views and (C) sagittal view using maximum intensity projection. (D) Volume rendering technique 3-dimensional reconstruction imaging in posterior view. LPV = left pulmonary vein; PV = portal vein; RA = right atrium; RPV = right pulmonary vein; SVC = superior vena cava; VV = vertical vein.

**Figure 3:**
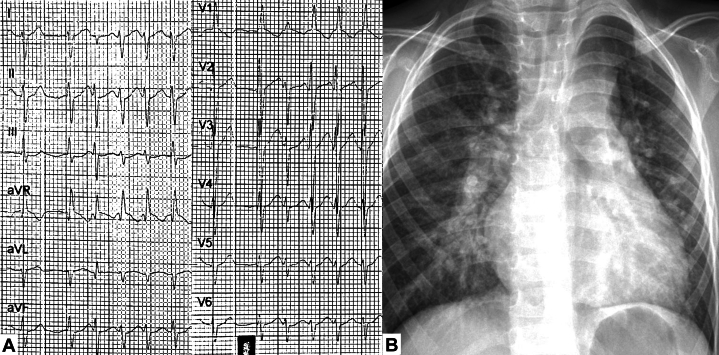
Patient 2 With Dual Drainage Total Anomalous Pulmonary Venous Connection (A) Initial electrocardiogram: RV hypertrophy, supraventricular extrasystoles with volume overload of RV. (B) Admission chest radiograph: biventricular cardiomegaly, with prominent bilateral pulmonary hila. RV = right ventricle.

**Figure 4:**
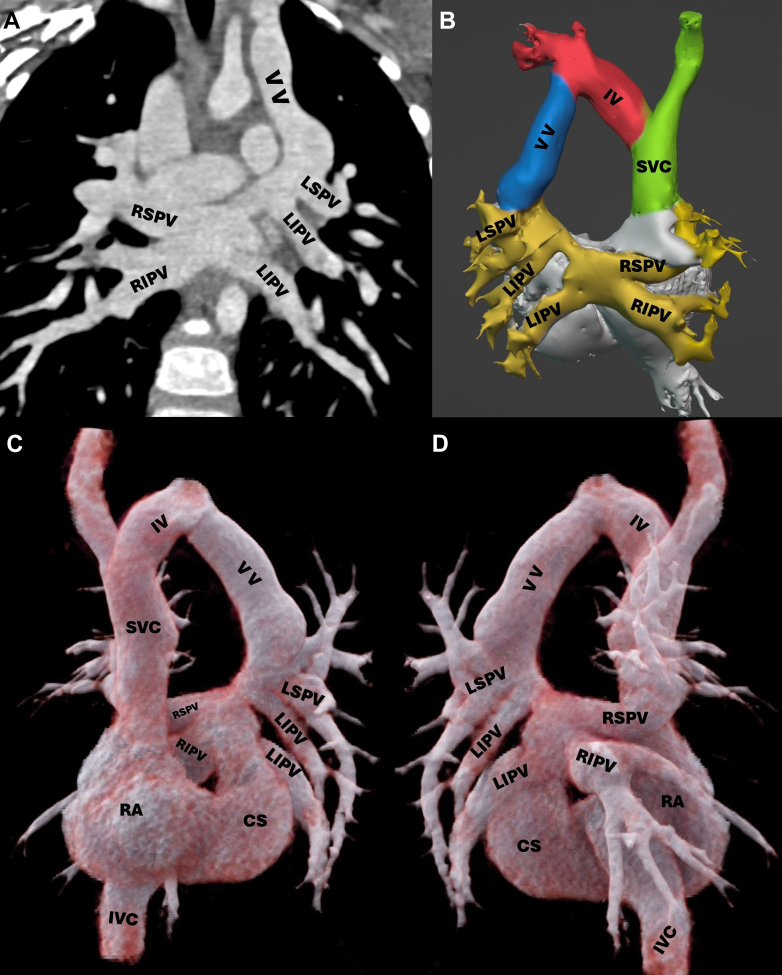
Patient 2 With Dual Drainage Total Anomalous Pulmonary Venous Connection CCTA showing drainage of the right and left pulmonary veins to a common collector, which flows to the coronary sinus and SVC via a VV connected to the innominate vein. (A) Coronal with MIP view. (B) VRT 3D reconstruction imaging in posterior view. (C, D) Cinematic VRT 3D reconstruction imaging in anterior and posterior views, respectively. 3D = 3-dimensional; CCTA = cardiac computed tomography angiography; CS = coronary sinus; IV = innominate vein; IVC = inferior vena cava; LIPV = left inferior pulmonary vein; LSPV = left superior pulmonary vein; MIP = maximum intensity projection; RA = right atrium; RIPV = right inferior pulmonary vein; SVC = superior vena cava; VRT = volume rendering technique; VV = vertical vein.

**Figure 5:**
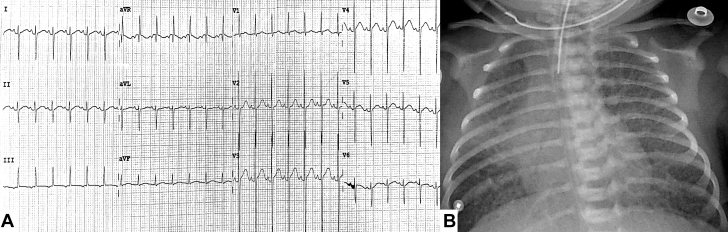
Patient 3 With Dual Drainage Total Anomalous Pulmonary Venous Connection (A) Initial electrocardiogram: RV hypertrophy with volume overload. (B) Admission chest radiograph: right heart enlargement with bilateral pulmonary infiltrates and prominent, plump pulmonary arteries. RV = right ventricle.

**Figure 6:**
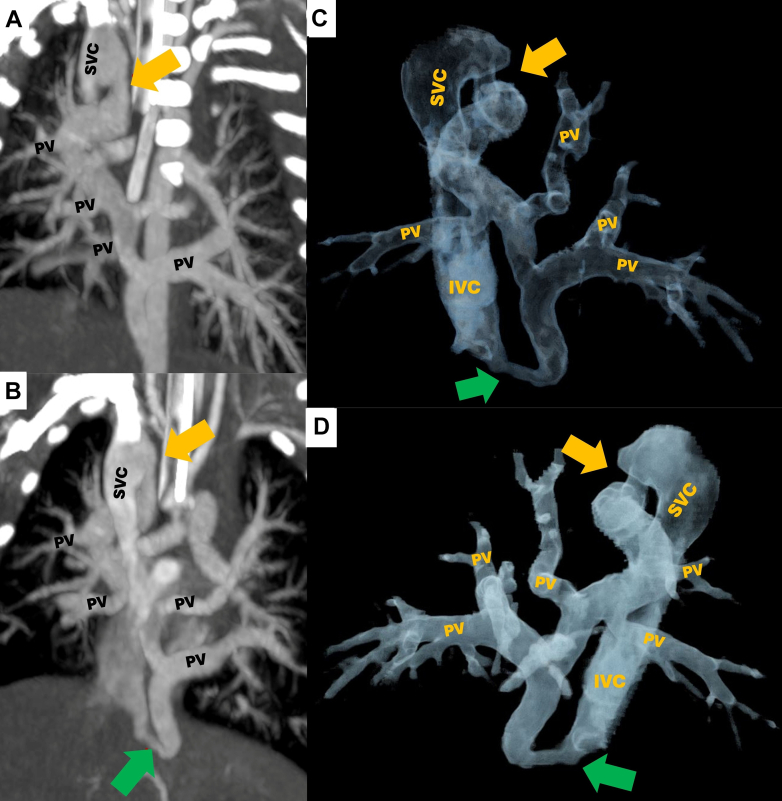
Patient 3 With Dual Drainage Total Anomalous Pulmonary Venous Connection Cardiac computed tomography angiography showing the right and left PVs draining independently into a retrocardiac collector with dual drainage: one branch to the SVC (yellow arrow) and the other (green arrow) to the confluence of the suprahepatic veins. (A, B) Coronal views using maximum intensity projection, (C) VRT 3D reconstruction in anterior view, (D) cinematic VRT 3D reconstruction imaging in posterior view. 3D = 3-dimensional; IVC = inferior vena cava; PV = pulmonary vein; SVC = superior vena cava; VRT = volume rendering technique.

Three patients with suspected CHD were admitted for further evaluation. Patients underwent initial clinical assessment and a transthoracic echocardiogram (TTE) along with cardiac computed tomography angiography (CCTA). Table 1 and Figure 1, Figure 2, Figure 3, Figure 4, Figure 5, Figure 6 describe the findings.

**Table 1:** Clinical and Echocardiographic Evaluation of Infants With Dual Drainage Total Anomalous Pulmonary Venous Connection

	Patient 1	Patient 2	Patient 3
Clinical features			
Family history of CHD	None	None	None
Vital signs on admission			
HR (beats/min)	160	90	151
BP (mm Hg)	81/62	87/67	81/45
Oxygen saturation (%)	60	81	80
Electrocardiogram on admission	Sinus rhythm	Sinus rhythm	Sinus rhythm
Findings on physical examination	Fixed split S2 heart sound. Pulmonary crackles	Nail clubbing. Continuous murmur at the left superior sternal border. Fixed split S2 heart sound	Pulmonary crackles. Continuous murmur at the left superior sternal border. Fixed split S2 heart sound
Echocardiogram			
LVEF (%)	74	67	55
RV FAC (%)	26	68	50
Valvulopathies	Mild tricuspid regurgitation and mild pulmonary regurgitation	Mild mitral regurgitation, mild mitral regurgitation, and mild pulmonary regurgitation	Mild tricuspid regurgitation
Associated anomalies	Secundum ASD	Secundum ASD and patent ductus arteriosus	Secundum ASD, patent ductus arteriosus, aortic arch hypoplasia, and left hypoplastic ventricle

ASD = atrial septal defect; BP = blood pressure; CHD = congenital heart disease; HR = heart rate; LVEF = left ventricular ejection fraction; RV FAC = right ventricular fractional area change.

### Case 1

A 3-month-old male infant with a history of multiple hospitalizations for respiratory failure was readmitted because of another episode that required mechanical intubation (Figure 1). TTE revealed a TAPVC with an atrial septal defect (ASD). CCTA showed a TAPVC with dual drainage via a common confluence with the portal vein, as well as through a vertical vein that drained into the superior vena cava (SVC) (Figure 2). Given the TAPVC anatomy and its associated defects, the Heart Team decided to perform emergency anastomosis of the lower collector to the left atrium, ligation of the vertical vein, and ASD closure, which was performed without complications. On follow-up 11 months later, the patient was asymptomatic.

### Case 2

A 3-year-old male toddler with progressive effort-related dyspnea was detected with a heart murmur (Figure 3). TTE revealed a TAPVC, ASD, and a patent ductus arteriosus (PDA). CCTA showed a TAPVC with dual drainage to the innominate vein and coronary sinus via a common confluence (Figure 4). The Heart Team decided to perform elective surgical correction involving the division and suturing of the vertical vein, ASD closure, coronary sinus unroofing, and PDA ligation, which was accomplished 6 months later without complications. On follow-up 5 years later, the patient was asymptomatic, allowing him to perform his activities.

### Case 3

A 9-day-old male newborn experienced respiratory failure at birth, requiring mechanical ventilation and vasopressor support (Figure 5). Initial TTE showed a TAPVC, ASD, and PDA associated with hypoplastic left ventricle and aortic arch hypoplasia. CCTA confirmed previous echocardiogram findings, which showed a TAPVC with dual drainage via a retrocardiac collector to the SVC and the confluence of the suprahepatic veins (Figure 6). Considering the disease's complexity and poor hemodynamic condition, the heart team opted for palliative care rather than surgery. The patient died a few days later.

## Discussion

“*Within mixed anomalous connections, in very rare situations, all of the pulmonary veins can join a common confluence, but the confluence itself then drains via separate channels, for example, to both a left vertical vein and the coronary sinus.”* — R.H. Anderson^1^

In Darling's classification of TAPVC, type IV or mixed type is distinguished by lack of confluence of the PVs into a collecting vein.^6^^,^^7^ Dual drainage TAPVC is a rare variant in which all 4 PVs enter a common venous chamber and drain into the systemic veins via 2 or more channels at the supracardiac, cardiac, or infracardiac levels.^4^ In our patients, all PVs were drained through a common collector with simultaneous drainage at 2 different cardiac levels, fulfilling the criteria for dual drainage TAPVC.

In this pathology, the embryological origin is the involution of the cardinal and umbilicovitelline veins of the splanchnic plexus, which initially drain pulmonary venous blood. Total anomalous drainage is caused by atresia or lack of development of the common PV and the persistence of the connection with the cardinal, umbilical, or vitelline venous systems.^7^

Patients with TAPVC usually present during the first few weeks of life with severe pulmonary venous hypertension, which makes them less likely to survive past the neonatal period without corrective surgery.^8^ The clinical presentation of these patients may include cyanosis, dyspnea, pulmonary congestion, or recurring lung infections;^5^^,^^7^ the condition is fatal without a connection to the left heart chambers, like an ASD or PDA.^7^ This case series consists of patients who developed respiratory symptoms at an early age and were referred for treatment. All cases had associated defects, with ASD being the most prevalent.

Although echocardiography provides sufficient information for diagnosis, other imaging modalities, such as cardiac magnetic resonance and CCTA, may also accurately describe pulmonary venous drainage.^6^^,^^9^ This case series highlights how cross-sectional imaging with multiplanar reconstruction, as provided by CCTA, allowed for the accurate type identification of TAPVC, including drainage separation in mixed variants.^9^

The goal of surgery in patients with TAPVC is to create a wide and nonrestrictive connection between all PVs and the left atrium.^6^ Failure to identify the dual drainage before surgery may impede the complete redirection of the PVs, which may lead to residual shunting postoperatively.^9^^,^^10^ The correct preoperative diagnosis and anatomic description of TAPVC are essential for surgical treatment planning.^6^ CCTA's high spatial resolution and multiplanar and 3-dimensional reconstruction capabilities make it an essential noninvasive diagnostic tool for the management and diagnosis of TAPVC. This imaging modality has become increasingly relevant in surgical planning for patients with complex CHD.

Although mortality after TAPVC repair has decreased, it remains higher among young patients and those with cardiac connection types or pulmonary venous obstructions despite improved perioperative care. In the case of mixed types of TAPVC, surgical mortality remains high, particularly in patients with complex patterns of pulmonary venous connections.^6^

We conducted a literature review on PubMed using the search terms TAPVC and dual/double drainage. The search only included full-text English articles; however, this search yielded only a few case reports, limited to one country (India), highlighting the rarity of this condition.

Table 2 summarizes the cases. Overall, most patients were young males who presented with respiratory symptoms. ASD is the most common associated defect. Based on the drainage patterns observed, the majority had supracardiac and cardiac drainage, with the SVC and coronary sinus being the most frequently involved drainage sites. Among cases with available outcome data, the mortality rate for surgical repair of TAPVC remains high. All these reported cases had substantial similarities to the present case series.

**Table 2:** Summary of Literature Review Cases of Dual Drainage TAPVC

First Author	Country	Patient Demographics	Presentation	Sites of Anomalous Drainage	Associated Defects	Treatment	Outcome
Siddharth et al^4^	India	26-year-old man	Exertional dyspnea	• Supracardiac: left vertical vein to innominate vein • Cardiac: coronary sinus	Secundum ASD	Surgical repair of TAPVC, ASD closure	Alive
Ojha et al^5^	India	4-year-old boy	Recurrent chest infections	• Supracardiac: vertical vein to left brachiocephalic vein, draining into right SVC • Cardiac: coronary sinus	Nonrestrictive ASD	NR	NR
Agrawal et al^6^	India	9-month-old boy	Tachypnea and desaturation	• Supracardiac: vertical vein to innominate vein, draining into right SVC • Cardiac: coronary sinus	Secundum ASD	Surgical correction of TAPVC	Alive
Faisal et al^8^	India	5-month-old boy	Cyanosis and respiratory distress	• Supracardiac: vertical vein to right SVC • Infracardiac: portal vein	Nonrestrictive ASD	Surgical repair	Died
Sinha et al^9^	India	7-year-old child	Cyanosis and failure to thrive	• Supracardiac: vertical vein into the left brachiocephalic vein to right SVC • Cardiac: coronary sinus	ASD, hypoplastic right left atrium	NR	NR
Nagulakonda et al^10^	India	6-week-old boy	Cyanosis and feeding difficulties	• Supracardiac: vertical vein into the left brachiocephalic vein to right SVC • Cardiac: coronary sinus	ASD	NR	NR
Present case series	Mexico	3-month-old boy	Respiratory failure	• Supracardiac: right vertical vein connected to SVC • Infracardiac: portal vein	ASD	Surgical repair of TAPVC, ASD closure	Alive
		3-year-old boy	Progressive effort dyspnea	• Supracardiac: innominate vein to SVC • Cardiac: coronary sinus	ASD, PDA	Surgical repair of TAPVC, ASD closure, and PDA ligation	Alive
		9-day-old boy	Respiratory failure	• Supracardiac: collector to SVC • Infracardiac: suprahepatic veins	ASD, PDA, aortic arch hypoplasia, left hypoplastic ventricle	Palliative care	Died

ASD = atrial septal defect; NR = no reported; PDA = patent ductus arteriosus; SVC = superior vena cava; TAPVC = total anomalous pulmonary venous connection.

## Conclusions

Dual drainage TAPVC is a very rare variant of mixed-type TAPVC with scarce reports in the literature. CCTA is a valuable tool for accurately determining the precise anatomy of TAPVCs with dual drainage, allowing for surgical planning for complete PV rerouting, repair of associated defects, and postoperative follow-up to detect complications.

## Funding Support and Author Disclosures

The authors have reported that they have no relationships relevant to the contents of this paper to disclose.
